# A Qualitative Textual Analysis of Feedback Comments in ePortfolios: Quality and Alignment with the CanMEDS Roles

**DOI:** 10.5334/pme.1050

**Published:** 2023-12-22

**Authors:** Sofie Van Ostaeyen, Mieke Embo, Tijs Rotsaert, Orphée De Clercq, Tammy Schellens, Martin Valcke

**Affiliations:** 1 Department of Educational Sciences at Ghent University in Belgium, Belgium; 2 Department of Nursing and Midwifery at the University of Antwerp, Belgium; 3 Department of Educational Sciences at Ghent University and in the Expertise Network Health and Care at the Artevelde University of Applied Sciences in Belgium, Belgium; 4 Language and Translation Technology Team at Ghent University in Belgium, Belgium

## Abstract

**Introduction:**

Competency-based education requires high-quality feedback to guide students’ acquisition of competencies. Sound assessment and feedback systems, such as ePortfolios, are needed to facilitate seeking and giving feedback during clinical placements. However, it is unclear whether the written feedback comments in ePortfolios are of high quality and aligned with the current competency focus. Therefore, this study investigates the quality of written feedback comments in ePortfolios of healthcare students, as well as how these feedback comments align with the CanMEDS roles.

**Methods:**

A qualitative textual analysis was conducted. 2,349 written feedback comments retrieved from the ePortfolios of 149 healthcare students (specialist medicine, general practice, occupational therapy, speech therapy and midwifery) were analysed retrospectively using deductive content analysis. Two structured categorisation matrices, one based on four literature-derived feedback quality criteria (*performance, judgment, elaboration* and *improvement*) and another one on the seven CanMEDS roles (*Medical Expert, Communicator, Collaborator, Leader, Health Advocate, Scholar* and *Professional*), guided the analysis.

**Results:**

The minority of the feedback comments (n = 352; 14.9%) could be considered of high quality because they met all four quality criteria. Most feedback comments were of moderate quality and met only two to three quality criteria. Regarding the CanMEDS roles, the *Medical Expert* role was most frequently represented in the feedback comments, as opposed to the roles *Leader* and *Health Advocate*.

**Discussion:**

The results highlighted that providing high-quality feedback is challenging. To respond to these challenges, it is recommended to set up individual and continuous feedback training.

## Introduction

In healthcare education, societal pressures have driven a shift to competency-based education (CBE), in which graduate outcomes are formulated as competencies and are aligned with the roles graduates will play in the workplace [1]. A range of competency frameworks is available to organise competencies in a coherent manner [1]. The Canadian Medical Education Directions for Specialists (CanMEDS) competency framework is widely used and has been validated in the context of different healthcare professions [2]. The CanMEDS framework clusters competencies which are required by a competent professional into seven roles: *Medical Expert, Communicator, Collaborator, Leader, Health Advocate, Scholar* and *Professional* [3]. Students have great opportunity during clinical placements students to engage in these roles and to achieve the underlying competencies [4,5].

From a CBE perspective it is of crucial importance that during these clinical placements students receive high-quality feedback [6]. Therefore, sound assessment systems that facilitate the delivery of high-quality feedback are required [6]. This explains the adoption of ePortfolios, which support students in seeking feedback and teachers and clinical mentors in giving feedback [7]. A written feedback comment collected in an ePortfolio can be defined as “specific information about the comparison between a trainee’s observed performance and a standard, given with the intent to improve the trainee’s performance.” [8, p.193] As a result, written feedback comments can be considered valuable and valid data sources for students, teachers and clinical mentors. For students, written feedback comments are expected to guide their self-regulated learning process [9]. For teachers and clinical mentors, written feedback comments facilitate identifying struggling students and enable early remediation [10]. Furthermore, these written feedback comments support teachers and clinical mentors when making trustworthy and defensible decisions about the student’s progress [11].

For written feedback comments to be valuable for students, teachers and clinical mentors it is imperative that they are of high quality [12,13]. In accordance with van de Ridder et al.’s [8] definition of feedback, four distinct quality criteria can be used to characterize feedback comments as high-quality. Such feedback comments contain (1) specific information about the student’s *performance* [14], (2) a *judgment* about that performance [14], (3) *elaboration* on why the performance was judged that way [15] and (4) guidance on how the student’s performance can be *improved* [16]. These four feedback quality criteria align closely with the widely recognized feedback model introduced by Hattie and Timperley [17]. This model articulates three essential questions that effective feedback addresses, representing distinct facets of feedback: (1) ‘Where am I going?’—pertaining to the *performance* criterion (feed-up); (2) ‘How am I going?’—relating to *judgment* and *elaboration* criteria (feed-back); and (3) ‘Where to next?’—focused on the *improvement* criterion (feed-forward). In order to obtain sufficiently rich written feedback comments to make valid decisions about student performance [18], it is imperative that feedback comments meet all four established quality criteria. Of course, the individual criteria can be present in subsections of feedback comments. However, when these subsections are combined, the four feedback quality criteria are expected to be represented.

In the context of CBE, an additional focus on feedback quality is needed that requires written feedback comments to develop a picture of the student’s competency acquisition [19]. Thus, written feedback comments must align with the roles and underlying competencies that graduating healthcare professionals should develop in view of providing effective and safe patient care, as specified in competency frameworks (e.g., CanMEDS framework) [20].

ePortfolios surged in popularity to support feedback provision during clinical placements with an emphasis on high-quality written feedback comments to establish CBE. However, empirical research about the quality of written feedback comments in ePortfolios is lacking. Few studies have investigated the quality of written feedback comments in students’ ePortfolios and have, to the best of our knowledge, only focused on feedback quality criteria [21,22,23]. The present study adopts a double focus by not only looking at the quality of written feedback comments in ePortfolios (1), but by also investigating how these written feedback comments are aligned with the seven CanMEDS roles (2). To achieve this, two research questions were addressed:

**RQ1**. What is the quality of written feedback comments in ePortfolios of healthcare students?

**RQ2**. How are the written feedback comments in these ePortfolios aligned with the CanMEDS roles?

## Methods

### Design

We conducted a qualitative, textual analysis study [24] by carrying out a retrospective analysis of written feedback comments collected in the ePortfolios of healthcare students during clinical placements. In this respect, we selected the constructivist research paradigm. Within this paradigm, it is argued that it is impossible for researchers to separate themselves from their beliefs and values [25]. Therefore, in the section ‘Reflexivity’ below, we describe our backgrounds to clarify how this may have influenced our interpretation and coding of the feedback comments.

### Ethical approval

Ethical approval was obtained from the Ethical Committee of the Faculty of Psychology and Educational Sciences of Ghent University (reference #2021-34) and a Data Transfer Agreement was signed between the Medbook company and Ghent University.

### Context

The study was conducted in Flanders (Belgium) and the feedback comments analysed were written in Dutch. Since feedback quality criteria and the CanMEDS roles are not dependent on the nature of a specific healthcare profession [2], we included different educational programs in this study. Five healthcare educational programs participated: specialist medicine (post-graduate), general practice (post-graduate), occupational therapy (undergraduate), speech therapy (undergraduate) and midwifery (undergraduate). As such, we were able to develop a general picture of the feedback comments in ePortfolios across educational programs, both in terms of quality and alignment with the CanMEDS roles.

The included educational programs implemented the same ePortfolio platform (Medbook) to support feedback provision during clinical placements. Because of the availability of a large number of feedback comments in Medbook’s database, we collaborated with the Medbook company to include the feedback comments as research data in this study.

### Data collection

The feedback comments used as research data in this study were written in the students’ ePortfolios in an open text box as feedback on a reflection (undergraduate programs) or as part of a (low-stakes) assessment (postgraduate programs). The frequency with which feedback comments were written varied by educational program. The data collection was conducted in June 2021.

To ensure participants’ privacy, we took three preventive measures. First, students’ explicit consent was required to participate in the study, which included allowing the Medbook company to share their feedback comments with the researchers. All students from the participating educational programs taking an internship during the academic year 2020–2021 were invited to participate. Second, this information was provided to students via a pop-up window in Medbook, ensuring that we did not need personal information to contact them. When a student logged into Medbook, this pop-up window appeared. The Medbook company tracked which students agreed to participate and then extracted their data from the Medbook database. Third, before providing the data to the researchers, the Medbook company replaced all names included in the feedback comments with the placeholder ‘[anonymised]’. We received one dataset for each educational program containing the feedback comments and the dates at which they were written.

### Data analysis

The feedback comments were analysed using deductive content analysis, a commonly used method for analysing written texts to describe and quantify phenomena (e.g. feedback comments) [26,27]. We followed the three phases of the content analysis process described by Elo and Kyngäs [27]: (1) preparation, (2) organising and (3) reporting.

#### Preparation phase

First, we selected the unit of analysis, which was a complete feedback comment extracted from the ePortfolios. Subsequently, we read through these feedback comments to familiarise ourselves with the data.

#### Organising phase

During the organising phase, we developed two structured categorisation matrices: one based on the four literature-derived quality criteria (*performance, judgment, elaboration* and *improvement*) and one based on the seven CanMEDS roles (*Medical Expert, Communicator, Collaborator, Leader, Health Advocate, Scholar* and *Professional)* with underlying competencies (see Appendix A and B). We obtained permission to use the CanMEDS framework from the Royal College of Physicians and Surgeons of Canada. To test the transparency of these categorisation matrices, they were used by three researchers (SVO, ME, HD), independently of one another, to code a pilot set of 30 feedback comments. Afterwards, the researchers discussed coding discrepancies and iteratively refined the categorisation matrices through four additional review rounds. After deciding on the final categorisation matrices, the feedback comments were coded in two stages.

In the first stage (July 2021), the feedback comments were coded in the annotation platform INCEpTION [28] using the four quality criteria. To ensure coding reliability, two researchers (SVO and SJA) coded 100 feedback comments independent of one another (see Table 1 for the Cohen’s Kappa values). Afterwards, these double-coded feedback comments were discussed to identify and resolve discrepancies. Here, special attention was paid to the criterion *elaboration* as the Cohen’s Kappa value for this code indicated fair agreement among the researchers [29]. We made minor changes to clarify differences between coding categories and identified exemplary instances that guided the subsequent coding. The first author then independently coded the complete set of feedback comments.

**Table 1:** Cohen’s Kappa values.


CODE	COHEN’S KAPPA VALUE

Quality criteria	

*Performance*	0.77

*Judgment*	0.68

*Elaboration*	0.24

*Improvement*	0.83

CanMEDS roles	

*Medical Expert*	0.63

*Communicator*	0.62

*Collaborator*	0.60

*Leader*	0.51

*Health Advocate*	0.50

*Scholar*	0.63

*Professional*	0.21


In the second stage (June 2022), the text coded with the quality criteria *performance, elaboration* and *improvement* was further enriched with the seven CanMEDS roles in Microsoft Excel (Office 365). The text coded with the *judgment* criterion was not considered because it referred to adjectives that per se cannot be related to the CanMEDS roles. We also ensured reliability in this stage by double-coding 100 feedback comments (SVO and OJ) (see Table 1 for the Cohen’s Kappa values). Discrepancies in the double-coded feedback comments were resolved through discussion. The roles *Professional, Collaborator, Leader* and *Health Advocate* were given careful attention because agreement among coders on these roles was fair to moderate [29]. Next, the first author coded independently all the feedback comments and subsequently discussed certain interpretations and uncertainties with the research team to reach a consensus.

#### Reflexivity

The first author, SVO, is a PhD student in Educational Sciences and is entirely independent from the educational programs in which the feedback comments were collected. The students had no personal connection with her. SVO tested the transparency of the categorisation matrices in collaboration with ME and HD. ME is a postdoctoral researcher with a background in midwifery and 20 years of experience as the coordinator of the midwifery program. HD is a researcher and also a midwife, providing her with firsthand experience in writing feedback comments to students. Their backgrounds offer unique perspectives on the quality of the feedback comments. SJA is a Master’s student in Educational Studies, and OJ is a PhD student in Educational Studies and Health Sciences. They received comprehensive training to conduct double coding. Throughout the process, discussions were held at various time points to address any uncertainties and discrepancies.

## Results

Of the 3,504 students who received the pop-up window in Medbook, 149 students gave consent to share the feedback comments in their ePortfolios. This resulted in a set of 2,349 feedback comments. Table 2 provides an overview of the number of students that gave consent and the number of analysed feedback comments for each educational program.

**Table 2:** Overview of the number of participating students and analysed feedback comments per educational program.


EDUCATIONAL PROGRAM	NO. OFSTUDENTS (%)	NO. OFFEEDBACK COMMENTS (%)

Specialistic medicine	47 (31.54)	649 (27.63)

General practice	62 (41.61)	256 (10.90)

Occupational therapy	9 (6.04)	229 (9.75)

Speech therapy	8 (5.37)	204 (8.68)

Midwifery	23 (15.44)	1,011 (43.04)

Total	149 (100)	2,349 (100)


### Quality of written feedback comments

During the first coding stage, we coded the feedback comments by considering the four quality criteria (See Appendix C for an example). Overall, most feedback comments fulfilled the criteria *performance* (n = 1,681; 71.56%), *judgment* (n = 1,613; 68.67%) and *improvement* (n = 1,298; 55.26%). The criterion *elaboration* was mostly lacking. This criterion was observed in less than a quarter of the feedback comments (n = 543; 23.12%).

Based on the number of coded quality criteria in a feedback comment these can be organised at three quality levels: low, moderate and high quality. We now describe the characteristics of the feedback comments from the different quality levels.

#### Low-quality feedback comments

Low-quality feedback comments met none (n = 342; 14.56%) or one (n = 347; 14.77%) quality criterion. Almost one-third of the feedback comments were of low quality (n = 689; 29.33%). Only a small number of the low-quality feedback comments contained information related to the *performance* (n = 27; 3.92%), the *judgment* (n = 8; 1.16%) or the *elaboration* criterion (n = 2; 0.29%). The criterion that was most often represented was *improvement* (n = 310; 44.99%), as illustrated in the following examples:

‘*Tighten skin when you insert the needle*.’ [comment_145, specialist medicine]

‘*Keep focus from start to finish. A small mistake can have big consequences*.’ [comment_516, specialist medicine]

#### Moderate-quality feedback comments

Feedback comments of moderate quality met two (n = 544; 23.16%) or three (n = 764; 32.52%) quality criteria. The analysis revealed that the quality of most of the feedback comments was moderate (n = 1,308; 55.68%). In contrast to low-quality feedback comments, almost all moderate-quality feedback comments applied the criteria *performance* (n = 1,302; 99.54%) and *judgment* (n = 1,253; 95.80%). Almost half of the feedback comments included information related to the criterion *improvement* (n = 636; 48.62%). The *elaboration* criterion was often lacking, as it was represented in only a few feedback comments (n = 189; 14.45%). The following feedback comments met the criteria *performance, judgment* and *improvement*, but missed information related to the *elaboration* criterion:

‘*Pleasant (non)verbal communication. Multiple problems in 1 consultation, handled this fairly flexibly. Make sure things are more prepared before they [the patients] are in the chair*.’ [comment_182, general practice]

‘*Wound care: Technique is known. Take more fluid and dare to clean the wound seam more thoroughly. Always hold the skin back when removing the sticker!!! Also again observe the patient more while acting*.’ [comment_1358, midwifery]

#### Hig-Hquality feedback comments

When feedback comments met all four quality criteria, they were of high quality. Only a minority of the feedback comments were rated as high quality (n = 352; 14.99%). The following examples of feedback comments met all four quality criteria:

‘*[anonymised] has been posting diabolos smoothly, also duravent and T-tube. Goes smoothly, doesn’t take long. Still enough attention to place the diabolo properly antero-inferiorly, though*.’ [comment_454, specialist medicine]

‘*Positive feedback from the supervising occupational therapist. You had prepared the activity well which also enabled you to differentiate. Tip: You can also organise this activity with fly swatters and a balloon*.’ [comment_712, occupational therapy]

### Alignment of written feedback comments with the CanMEDS roles

In the second coding stage, we further enriched the feedback comments with the CanMEDS roles. An example feedback comment for each CanMEDS role is provided in Appendix C.

The analysis indicated that the role *Medical Expert* was identified most frequently in the feedback comments (n = 1,530; 65.13%). The roles *Communicator* (n = 790; 33.63%), *Scholar* (n = 712; 30.31%), *Collaborator* (n = 634; 26.99%) and *Professional* (n = 375; 15.96%) were less frequently represented. The roles *Leader* (n = 256; 10.90%) and *Health Advocate* (n = 217; 9.24%) appeared in the least number of feedback comments.

Furthermore, it is important to note that a minor section of the feedback comments could not be related to any CanMEDS role (n = 354; 15.07%). Examples of such instances are:

‘*No remarks*’ [comment_169, specialist medicine]

‘*Practice makes perfect!*’ [comment_2, specialist medicine]

‘*Keep up the good work!*’ [comment_893, general practice]

## Discussion

To our knowledge, this is the first study to investigate the quality of written feedback comments in ePortfolios, as well as how these feedback comments can be aligned with the CanMEDS roles. The results revealed that the minority of the feedback comments could be considered of high quality. Most feedback comments met two or three quality criteria and were of moderate quality. Furthermore, the *Medical Expert* role was most frequently represented in the feedback comments, in contrast to the roles *Leader* and *Health Advocate*.

The results confirm observations about the critical quality of written feedback comments in healthcare education in general [30] and in ePortfolios in particular [21,23]. Most of the feedback comments contained information related to the criteria *performance, judgment* and *improvement*. However, the *elaboration* criterion was often lacking, which implies that crucial information making the feedback comments specific, was mostly missing. The paucity of specific feedback comments is a well-known phenomenon in healthcare education [14,23,31,32] and raises concerns about feedback providers’ readiness and skills to provide feedback. While acknowledging their responsibility to provide students with high-quality feedback to foster competency development and performance improvement, feedback providers in healthcare education face considerable challenges in meeting this demand [33,34]. The provision of high-quality feedback necessitates a diverse skill set among feedback providers [17]. Research reveals a deficiency in feedback skills among feedback providers in healthcare education [35], as they struggle to use feedback forms accurately, encounter difficulties in applying predefined learning outcomes as assessment criteria for students’ competencies and face challenges in providing high-quality feedback, even after receiving training [35,36,37]. As the provision of non-specific feedback comments has a negative impact on the student’s feedback-seeking behaviour [13], it is imperative for future research to investigate why feedback providers struggle to provide specific feedback to students and to identify the precise skills that require development to achieve feedback of a higher quality.

This study sheds new light on the representation of the CanMEDS roles in written feedback comments. The results show that most feedback was given in relation to the *Medical Expert* role. This can be explained because healthcare professionals consider this as the most important role; thus leading to a biased feedback orientation when assessing students [38]. The roles *Leader* and *Health Advocate* were represented to a lesser extent. This might be because these roles are underrepresented in healthcare curricula and learning activities during clinical placements [39,40]. Furthermore, teachers and clinical mentors know little about how to teach and assess these roles [41]. Although opportunities to learn about the *Leader* and *Health Advocate* role are optimal during clinical placements, they do not seem to be adequately reflected in feedback comments, which hinders related acquisition of these roles [42]. Given the inconsistent representation of certain CanMEDS roles in feedback comments [43], setting up training initiatives to improve awareness of the CanMEDS roles and to provide practical ideas and a shared language to incorporate the roles in feedback comments, is recommended [12,41,42].

Enhancing the quality of written feedback comments seems to be a Sisyphean task [44]. Training initiatives are available to train teachers and clinical mentors in writing high-quality feedback comments. Research about generic training initiatives – such as formal training programs and workshops – mirror inconsistent effects [31,45], mainly due to competing professional interests for time, including patient care, teaching, research, faculty promotion and administration [32]. As an alternative, a personalised approach could be adopted that focuses on giving feedback to feedback providers on their feedback comments and on coaching over a longer period of time [31]. Certainly in the context of ePortfolio use, an individual, continuous training approach is recommended [46]. However, the implementation of such a personal training approach in a traditional way (e.g., feedback on feedback from educational staff) is challenging due to its time and resource-intensive nature. Future research is required to explore how these time and resource constraints can be overcome.

The present study demonstrated that the manual analysis of written feedback comments is time-consuming. This is a growing problem as more and more digital feedback tools are being adopted in healthcare education, leading to increasing amounts of written feedback comments [47]. Recent technological advances in the field of artificial intelligence and Natural Language Processing (NLP) might be helpful to evaluate large amounts of feedback comments in a short amount of time [48]. Researchers already demonstrated the potential utility of NLP techniques to classify the quality of written feedback comments [47,48]. Future research should explore how these NLP techniques could be integrated into feedback tools in a way that they can provide real-time support to teachers and clinical mentors in writing feedback comments.

This study should be considered in light of its limitations. First, we only analysed written feedback comments. It is possible that more high-quality verbal feedback was provided to the students during formal or informal feedback conversations, but that this was not documented in the ePortfolios [49]. Given that this study aimed to investigate written feedback comments in ePortfolios, this does not impact the results. However, future research could explore whether the results of the current analysis are also applicable to verbal feedback. Secondly, we did not consider individual differences in student’s, teachers’ and clinical mentors’ characteristics. Mooney et al. [50], for example, found that feedback quality was associated with the feedback provider’s gender. Additionally, a within-subject variation is possible, depending on the feedback performance phase being focused upon by the same feedback provider. However, to ensure the participants’ privacy, this was not questioned when collecting the feedback comments. A third limitation is linked to the differences in sample sizes for each educational program. This can be explained by the timing of data collection. Due to an extended ethical approval process, data were collected later than initially planned (June 2021). This period coincided with the end of the academic year, resulting in fewer students who were still on their clinical placements and consequently fewer students opening their ePortfolios and viewing the pop-up window. Although this limitation is not a shortcoming for the present study, this could be an issue when subsequent research would focus on the quality of feedback comments in each educational program. The current dataset cannot be used to compare the feedback comments of different educational programs.

In conclusion, the results of this study highlighted that providing high-quality feedback that develops a picture of the student’s competency acquisition is challenging. The majority of the feedback comments were of moderate quality and focused on the student’s performance related to the *Medical Expert* role. To respond to these challenges, it is recommended to set up individual, continuous training initiatives. In this regard, the opportunities to use NLP techniques to offer real-time support to feedback providers in writing feedback should be explored.

## Data Accessibility Statement

The participants of this study did not give written consent for their data to be shared publicly, so due to the sensitive nature of the research supporting data is unavailable.

## Additional Files

The additional files for this article can be found as follows:

10.5334/pme.1050.s1 Appendix A. Structured categorisation matrix quality criteria.

10.5334/pme.1050.s2 Appendix B. Structured categorisation matrix CanMEDS roles.

10.5334/pme.1050.s3 Appendix C. Example feedback comment for each feedback quality criterion and CanMEDS role (translated from Dutch).
