# Supplementary material for: A Qualitative Textual Analysis of Feedback Comments in ePortfolios: Quality and Alignment with the CanMEDS Roles
Source: Perspect Med Educ. 2023 Dec 22;12(1):584–93. doi: 10.5334/pme.1050 (PMC10742175; doi:10.5334/pme.1050)
Supplement: Appendix A. — Structured categorisation matrix quality criteria. [file pme-12-1-1050-s1.pdf]

Appendix A. Structured categorisation matrix quality criteria

Code	Definition
Performance	The task on which the feedback is provided. This task must be observable.
Judgment	An adjective used to denote the gap between the performance and a standard. It is an evaluative statement expressed as a judgment on past performance.
Elaboration	Informative statement that builds further on the judgment expressed as a question, confirmation, suggestion, or justification… (e.g. it indicates what went well or did not go well, why it went well or not…). In doing so, it gives more details about the judgment.
Improvement	The aim of feedback is to improve the trainee’s performance. The feedback must suggest strategies for improvement.
