# Supplementary material for: A Qualitative Textual Analysis of Feedback Comments in ePortfolios: Quality and Alignment with the CanMEDS Roles
Source: Perspect Med Educ. 2023 Dec 22;12(1):584–93. doi: 10.5334/pme.1050 (PMC10742175; doi:10.5334/pme.1050)
Supplement: Appendix B. — Structured categorisation matrix CanMEDS roles. [file pme-12-1-1050-s2.pdf]

Appendix B. Structured categorisation matrix CanMEDS roles

Code	Definition	
Role	Key competencies	Enabling competencies
Medical expert	1. Practise medicine within their defined scope of practice and expertise	1.1 Demonstrate a commitment to high-quality care of their patients
		1.2 Integrate the CanMEDS Intrinsic Roles into their practice of medicine
		1.3 Apply knowledge of the clinical and biomedical sciences relevant to their discipline
		1.4 Perform appropriately timed clinical assessments with recommendations that are presented in an organised manner
		1.5 Carry out professional duties in the face of multiple, competing demands
		1.6 Recognise and respond to the complexity, uncertainty, and ambiguity inherent in medical practice
	2. Perform a patient-centred clinical assessment and establish a	2.1 Prioritise issues to be addressed in a patient encounter

	management plan	2.2 Elicit a history, perform a physical exam, select appropriate investigations, and interpret their results for the purpose of diagnosis and management, disease prevention, and health promotion
		2.3 Establish goals of care in collaboration with patients and their families, which may include slowing disease progression, treating symptoms, achieving cure, improving function, and palliation
		2.4 Establish a patient-centred management plan
	3. Plan and perform procedures and therapies for the purpose of assessment and/or management	3.1 Determine the most appropriate procedures or therapies
		3.2 Obtain and document informed consent, explaining the risks and benefits of, and the rationale for, a proposed procedure or therapy
		3.3 Prioritise a procedure or therapy, taking into account clinical urgency and available resources
		3.4 Perform a procedure in a skilful and safe manner, adapting to

		unanticipated findings or changing clinical circumstances
	4. Establish plans for ongoing care and, when appropriate, timely consultation	4.1 Implement a patient-centred care plan that supports ongoing care, follow-up on investigations, response to treatment, and further consultation
	5. Actively contribute, as an individual and as a member of a team providing care, to the continuous improvement of healthcare quality and patient safety	5.1 Recognise and respond to harm from healthcare delivery, including patient safety incidents
		5.2 Adopt strategies that promote patient safety and address human and system factors
Communicator	1. Establish professional therapeutic relationships with patients and their families	1.1 Communicate using a patient-centred approach that encourages patient trust and autonomy and is characterised by empathy, respect, and compassion
		1.2 Optimise the physical environment for patient comfort, dignity, privacy, engagement, and safety
		1.3 Recognise when the values, biases, or perspectives of patients, or other healthcare professionals may

		have an impact on the quality of care, and modify the approach to the patient accordingly
		1.4 Respond to a patient’s non-verbal behaviours to enhance communication
		1.5 Manage disagreements and emotionally charged conversations
		1.6 Adapt to the unique needs and preferences of each patient and to his or her clinical condition and circumstances
	2. Elicit and synthesise accurate and relevant information, incorporating the perspectives of patients and their families	2.1 Use patient-centred interviewing skills to effectively gather relevant biomedical and psychosocial information
		2.2 Provide a clear structure for and manage the flow of an entire patient encounter
		2.3 Seek and synthesise relevant information from other sources, including the patient’s family, with the patient’s consent
	3. Share healthcare information and plans with patients and their families	3.1 Share information and explanations that are clear, accurate,

		and timely, while checking for patient and family understanding
		3.2 Disclose harmful patient safety incidents to patients and their families accurately and appropriately
	4. Engage patients and their families in developing plans that reflect the patient’s healthcare needs and goals	4.1 Facilitate discussions with patients and their families in a way that is respectful, non-judgmental, and culturally safe
		4.2 Assist patients and their families to identify, access, and make use of information and communication technologies to support their care and manage their health
		4.3 Use communication skills and strategies that help patients and their families make informed decisions regarding their health
	5. Document and share written and electronic information about the medical encounter to optimise clinical decision-making, patient safety, confidentiality, and privacy	5.1 Document clinical encounters in an accurate, complete, timely, and accessible manner, in compliance with regulatory and legal requirements
		5.2 Communicate effectively using a written health record, electronic

		medical record, or other digital technology
		5.3 Share information with patients and others in a manner that respects patient privacy and confidentiality and enhances understanding
Collaborator	1. Work effectively with other colleagues in the healthcare professions	1.1 Establish and maintain positive relationships with other colleagues in the healthcare professions to support relationship-centred collaborative care
		1.2 Negotiate overlapping and shared responsibilities with other colleagues in the healthcare professions in episodic and ongoing care
		1.3 Engage in respectful shared decision-making with other colleagues in the healthcare professions
	2. Work with other colleagues in the healthcare professions to promote understanding, manage differences, and resolve conflicts	2.1 Show respect towards collaborators
		2.2 Implement strategies to promote understanding, manage differences, and resolve conflicts in a manner that supports a collaborative culture

	3. Hand over the care of a patient to another healthcare professional to facilitate continuity of safe patient care	3.1 Determine when care should be transferred to another healthcare professional
		3.2 Demonstrate safe handover of care, using both verbal and written communication, during a patient transition to a different healthcare professional, setting, or stage of care
Leader	1. Contribute to the improvement of healthcare delivery in teams, organisations, and systems	1.1 Apply the science of quality improvement to contribute to improving systems of patient care
		1.2 Contribute to a culture that promotes patient safety
		1.3 Analyse patient safety incidents to enhance systems of care
		1.4 Use health informatics to improve the quality of patient care and optimise patient safety
	2. Engage in the stewardship of healthcare resources	2.1 Allocate healthcare resources for optimal patient care
		2.2 Apply evidence and management processes to achieve cost-appropriate care
	3. Demonstrate leadership in professional practice	3.1 Demonstrate leadership skills to enhance healthcare

		3.2 Facilitate change in healthcare to enhance services and outcomes
	4. Manage career planning, finances, and health human resources in a practice	4.1 Set priorities and manage time to integrate practice and personal life
		4.2 Manage a career and a practice
		4.3 Implement processes to ensure personal practice improvement
Health advocate	1. Respond to an individual patient's health needs by advocating with the patient within and beyond the clinical environment	1.1 Work with patients to address determinants of health that affect them and their access to needed health services or resources
		1.2 Work with patients and their families to increase opportunities to adopt healthy behaviours
		1.3 Incorporate disease prevention, health promotion, and health surveillance into interactions with individual patients
	2. Respond to the needs of the communities or populations they serve by advocating with them for system-level change in a socially accountable manner	2.1 Work with a community or population to identify the determinants of health that affect them
		2.2 Improve clinical practice by applying a process of continuous quality improvement to disease

		prevention, health promotion, and health surveillance activities
		2.3 Contribute to a process to improve health in the community or population they serve
Scholar	1. Engage in the continuous enhancement of their professional activities through ongoing learning	1.1 Develop, implement, monitor, and revise a personal learning plan to enhance professional practice
		1.2 Identify opportunities for learning and improvement by regularly reflecting on and assessing their performance using various internal and external data sources
		1.3 Engage in collaborative learning to continuously improve personal practice and contribute to collective improvements in practice
	2. Teach students, residents, the public, and other healthcare professionals	2.1 Recognise the influence of role-modelling and the impact of the formal, informal, and hidden curriculum on learners
		2.2 Promote a safe learning environment
		2.3 Ensure patient safety is maintained when learners are

		involved
		2.4 Plan and deliver a learning activity
		2.5 Provide feedback to enhance learning and performance
		2.6 Assess and evaluate learners, teachers, and programs in an educationally appropriate manner
	3. Integrate best available evidence into practice	3.1 Recognise practice uncertainty and knowledge gaps in clinical and other professional encounters and generate focused questions that address them
		3.2 Identify, select, and navigate pre-appraised resources
		3.3 Critically evaluate the integrity, reliability, and applicability of health related research and literature
		3.4 Integrate evidence into decision-making in their practice
	4. Contribute to the creation and dissemination of knowledge and practices applicable to health	4.1 Demonstrate an understanding of the scientific principles of research and scholarly inquiry and the role of research evidence in healthcare
		4.2 Identify ethical principles for

		research and incorporate them into obtaining informed consent, considering potential harms and benefits, and considering vulnerable populations
		4.3 Contribute to the work of a research program
		4.4 Pose questions amenable to scholarly inquiry and select appropriate methods to address them
		4.5 Summarise and communicate to professional and lay audiences, including patients and their families, the findings of relevant research and scholarly inquiry
Professional	1. Demonstrate a commitment to patients by applying best practices and adhering to high ethical standards	1.1 Exhibit appropriate professional behaviours and relationships in all aspects of practice, demonstrating honesty, integrity, humility, commitment, compassion, respect, altruism, respect for diversity, and maintenance of confidentiality
		1.2 Demonstrate a commitment to excellence in all aspects of practice
		1.3 Recognise and respond to ethical

		issues encountered in practice
		1.4 Recognise and manage conflicts of interest
		1.5 Exhibit professional behaviours in the use of technology-enabled communication
	2. Demonstrate a commitment to society by recognising and responding to societal expectations in healthcare	2.1 Demonstrate accountability to patients, society, and the profession by responding to societal expectations of other healthcare professionals
		2.2 Demonstrate a commitment to patient safety and quality improvement
	3. Demonstrate a commitment to the profession by adhering to standards and participating in healthcare professional-led regulation	3.1 Fulfil and adhere to the professional and ethical codes, standards of practice, and laws governing practice
		3.2 Recognise and respond to unprofessional and unethical behaviours in other colleagues in the healthcare professions
		3.3 Participate in peer assessment and standard-setting
	4. Demonstrate a commitment to healthcare professional health and	4.1 Exhibit self-awareness and manage influences on personal well-

	well-being to foster optimal patient care	being and professional performance
		4.2 Manage personal and professional demands for a sustainable practice throughout the healthcare professional life cycle
		4.3 Promote a culture that recognises, supports, and responds effectively to colleagues in need
