# Supplementary material for: A Qualitative Textual Analysis of Feedback Comments in ePortfolios: Quality and Alignment with the CanMEDS Roles
Source: Perspect Med Educ. 2023 Dec 22;12(1):584–93. doi: 10.5334/pme.1050 (PMC10742175; doi:10.5334/pme.1050)
Supplement: Appendix C. — Example feedback comment for each feedback quality criterion and CanMEDS role (translated from Dutch). [file pme-12-1-1050-s3.pdf]

Appendix C. Example feedback comment for each feedback quality criterion and CanMEDS role (translated from Dutch)

Quality criterion	Example feedback comment
Performance<br>*Judgment*<br>*Elaboration*<br>*Improvement*	[Blood sampling]$^{\textit{performance}}$ went [well]$^{\textit{judgment}}$, [good preparation, sterile procedure, smoothly punctured]$^{\textit{elaboration}}$. [Indeed, remove your tube first. Continue practicing venipuncture in babies.]$^{\textit{improvement}}$ [Your technique]$^{\textit{performance}}$ is [good]$^{\textit{judgment}}$! [comment_1026, midwifery]

CanMEDS role	Example feedback comment
Medical Expert	*'The blood collection went well. I saw that you fixed the needle nicely when changing the tubes. The procedures went smoothly and your aftercare was also correct.'* [comment_2254, midwifery]
Communicator	*'You are indeed very calm while taking the test, which is very pleasant for the children. [anonymised] was a bit uncomfortable in the beginning but you handled that well.'* [comment_1183, speech therapy]
Collaborator	*'Handover was good, SBAR structured.'* [comment_128, specialist medicine]
Leader	*'... Correctly took charge of a situation within our field. There were clear instructions for employees. These could indeed be structured a little better. But in acute situations, it is never evident to follow a nice structure. Well done!'*

	[comment_362, specialist medicine]
Health Advocate	*'… You explained the health information related to Menopur very well. You demonstrated how she can do her injection herself and explained it at the patient's level…'* [comment_1363, midwifery]
Scholar	*'It is notable that every unprecedented detail is explored. She misses no opportunity to gather additional information. In doing so, she knows which sources to consult. She is open to feedback.'* [comment_1034, general practice]
Professional	*'… Yet you will not think you are able to do everything already. When in doubt about vaginal examination or rupture of membranes, you will spontaneously say that you are not very sure. And whether we want to check. This makes us trust you 100%.'* [comment_1156, midwifery]
